# Construction of a lactate-related prognostic signature for predicting prognosis after surgical repair for acute type a aortic dissection

**DOI:** 10.3389/fphys.2022.1008869

**Published:** 2022-11-16

**Authors:** Zhigang Wang, Kai Li, Jingfang Xu, Xiaofeng Cheng, Dongjin Wang

**Affiliations:** ^1^ Department of Cardio-thoracic Surgery, Affiliated Drum Tower Hospital, Medical School of Nanjing University, Nanjing, China; ^2^ Department of Nephrology, Nanjing Drum Tower Hospital Clinical College of Nanjing University of Chinese Medicine, Nanjing, China; ^3^ Department of Cardio-thoracic Surgery, Nanjing Drum Tower Hospital Clinical College of Traditional Chinese and Western Medicine, Nanjing University of Chinese Medicine, Nanjing, China

**Keywords:** aortic dissection, 30-day mortality, lactate, intensive care unit, risk factor, outcome

## Abstract

**Background:** Serum lactate is commonly measured in the perioperative period in patients who have undergone surgery for an acute type A aortic dissection (ATAAD). However, conflicting data has been reported as to whether lactate elevation is associated with short-term prognosis. The aim of the current study was to determine the association between perioperative arterial lactate levels and postoperative 30-day mortality.

**Methods:** Patients who underwent repair of a ATAAD at our institution were retrospectively screened and those with comprehensive measurements of serum lactate before surgery and at 0, 1, 3, 6, 12, and 24 h after surgery in the intensive care unit (ICU) were selected for the analysis. Patients’ demographic features and outcomes were reviewed to determine risk factors associated with 30-day mortality using logistic regression modeling. The association between serum lactate levels at different time points and 30-day mortality were analyzed by receiver-operating characteristic curves.

**Results:** 513 patients were identified and retrospectively analyzed for this study including 66 patients (12.9%) who died within 30 days after surgery. Patients who died within 30 days after surgery had elevated lactate levels measured before surgery and at 0, 1, 3, 6, 12, and 24 h after their ICU stay. Lactate measured at 24 h post ICU admission (odds ratio, 2.131; 95% confidence interval, 1.346–3.374; p = 0.001) was a predictor of 30-day mortality. The area under the curve (AUC) for 30-day mortality with lactate levels at 12 h and 24 h post ICU stay were 0.820 and 0.805, respectively.

**Conclusion:** Early elevation of lactate level is correlated with increased 30-day mortality in patients who received ATAAD surgical repair.

## Introduction

Acute type A aortic dissection (ATAAD) is a lethal disease with an in-hospital mortality ranging from 16% to 83% (Kilic et al., 2013). Despite recent advances in endovascular interventions and postoperative management, open surgery remains the gold standard for the treatment of ATAAD. A variety of risk factors for in-hospital mortality in ATAAD have been reported (Pansini et al., 1998; Santini et al., 2007). However, many other factors may contribute to this devastating outcome.

Serum lactate has now been shown to be associated with inflammation, autoimmunity, and cancer (Pucino et al., 2017; Certo et al., 2021; Manoharan et al., 2021). Recent evidence suggests that lactate accumulates at sites of chronic inflammation and that these levels can increase *via* metabolic reprogramming (Pucino et al., 2019). Clinically, it has been known that lactate levels can be used as a surrogate to measure tissue perfusion; and its elevation has been shown to be associated with an increased risk of death in infection, sepsis, trauma, and patients undergoing open surgical procedures (Nguyen et al., 2004; Ding et al., 2018; Ilias et al., 2018).

The association between elevated lactate levels and increased mortality has been well studied in cardiac surgery (Kogan et al., 2012; Hajjar et al., 2013; Renew et al., 2016; Bennett et al., 2017). However, conflicting results have been reported for its prognostic value to predict mortality in patients undergoing surgical repair of an ATAAD (Bennett et al., 2017; Zindovic et al., 2018; Gemelli et al., 2022). In addition, the lactate levels used in most studies were obtained at a single time point; while recent studies have indicated that serial lactate measurements, rather than one-time measurements, are more reliable for assessing risk and outcomes (Lazzeri et al., 2015; Li et al., 2015). Therefore, this study sought to examine the correlation between serial levels of serum lactate and 30-day mortality in patients who underwent ATAAD surgical repair.

## Materials and methods

### Study design

This study was a single-center, retrospective cohort study enrolling consecutive patients who received emergency surgical repair of an ATAAD between January 2019 and December 2020 at the Nanjing Drum Tower Hospital. Patients who died within 24 h after surgery and with incomplete medical records were excluded. All patients were divided into a non-survivor group and a survivor group based on their 30-day mortality. This study was approved by the Ethics Committee of Nanjing Drum Tower Hospital with a waiver of informed consent considering the retrospective nature of this study (Approved number: 2022-084-01).

### Data collection and definition

Patients’ clinical data were extracted from the institutional electronic medical record system. All patients were transferred to the intensive care unit (ICU) after completion of surgery. Acute kidney injury was diagnosed according to the Kidney Disease Improving Global Outcomes criteria (Kellum et al., 2013). Prolonged mechanical ventilation was defined as total ventilation time exceeded 48 h in the postoperative period. Malperfusion was defined as the presence of the following conditions before surgery: cardiac malperfusion with ST changes, echocardiographic signs of myocardial ischemia, or CK-MB level >75 mmol/L; cerebral malperfusion with loss of lateralized central neurological function; gastrointestinal malperfusion with mesenteric or liver ischemia; peripheral malperfusion with absent pulses or loss of sensory or motor function of the upper or lower extremities; spinal malperfusion with transient or permanent paraplegia (Zindovic et al., 2018). Shock was defined as systolic blood pressure less than 90 mmHg, regardless of the etiology.

### Lactate

Arterial lactate levels were measured as part of a comprehensive blood gas analysis preoperatively and at 0, 1, 3, 6, 12, and 24 h after ICU admission. Addition measurements were taken for patients with unstable conditions. All lactate measurements were examined with the ABL Flex 800 system (Radiometer Medical, Copenhagen, Denmark) with a normal reference of 1.0–1.8 mmol/L.

### Surgical procedures

All procedures were performed *via* a median sternotomy. Briefly, the femoral or right axillary artery was dissected for arterial inflow for cardiopulmonary bypass (CPB). Surgical repair techniques were chosen depending on the location of the intimal tear and the extent of the dissection. A root reinforcement reconstruction was routinely performed for the proximal segment. An aortic valve replacement or Bentall procedure was performed when the dissection involved the coronary ostia or aortic valve, or in the presence of an aortic root aneurysm. Coronary artery bypass grafting procedure was performed in the following scenarios: cardiac malperfusion upon admission or the surgeons’ choice according to the patients’ coronary artery status during the operation.

### Statistical analysis

Categorical variables were presented as the counts and percentages and were compared using the χ^2^ test or the Fisher’s exact test, as appropriate. Continuous variables were compared with the student’s *t*-test or the Mann–Whitney *U*-test and were presented as mean ± standard deviation for normal distributed variables or medians (interquartile ranges, 25–75th percentile) for variables not following normal distribution. The discriminatory performance of lactate as a predictor of mortality was determined using a receiver operating characteristic (ROC) curve. The area under the ROC curve was calculated to quantify the accuracy of lactate levels in predicting 30-day mortality. To estimate cut off values, Youden-Index J was calculated by the following equitation: J = sensitivity + (specificity-1). Cut off values were determined by the highest Youden-Index.

Univariable and multivariable logistic regression models were used to identify risk factors for 30-day mortality (stepwise forward method for multivariable regression analysis). Covariates that were either statistically significant on univariable analysis or considered clinically important were included in multivariable models. All probability values were two-tailed, and *p* values <0.05 were considered statistically significant. Statistical analyses were performed using the SPSS software, version 26 (IBM, Armonk, NY, United states).

## Results

### Demographic characteristics

**FIGURE 1:**
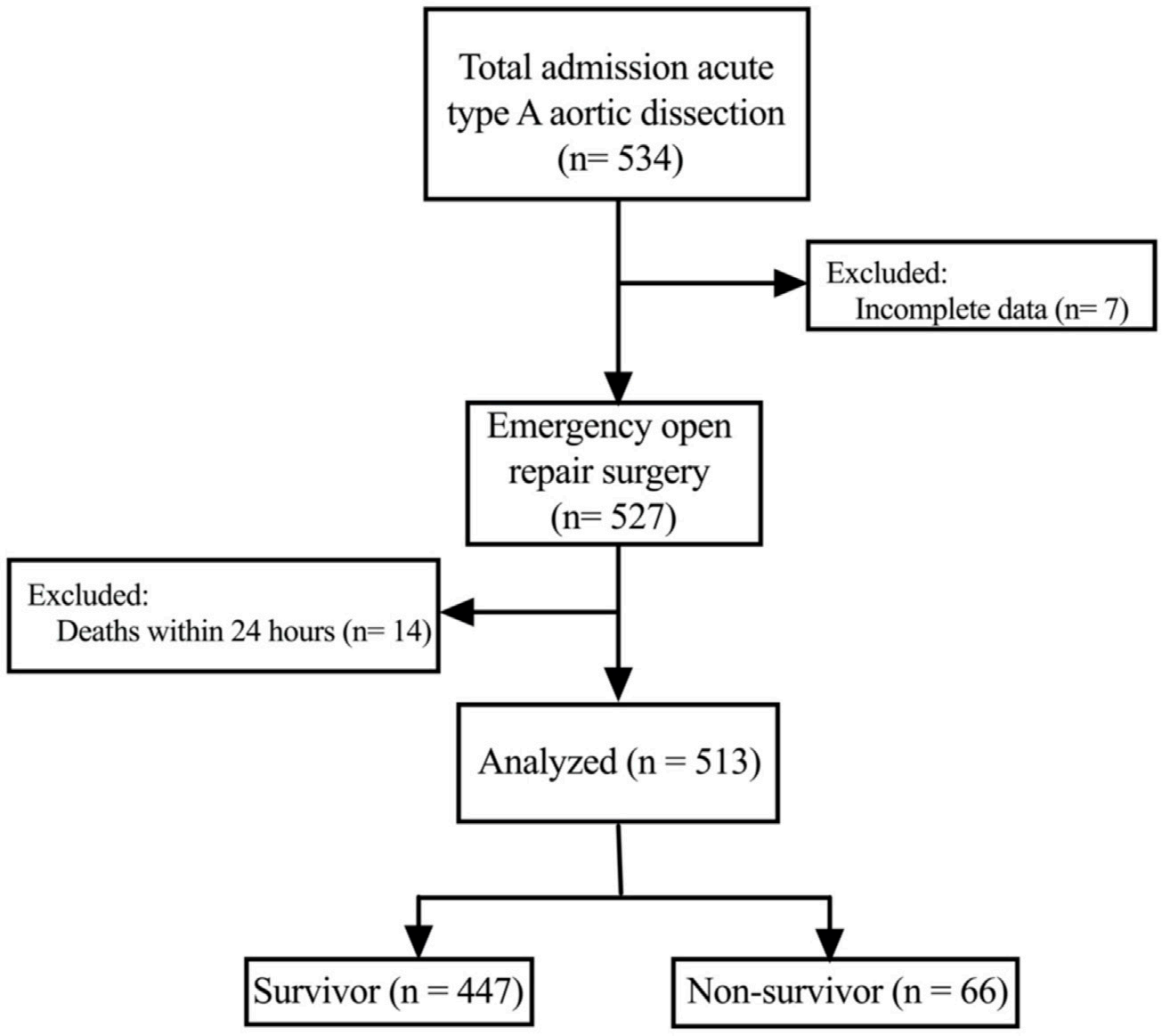
Flowchart of the patient population.

As shown in Figure 1, a total of 513 patients were included in this analysis. The mean age of the patients was 54.1 ± 13.5 years, and most were males. The 30-day mortality of all patients was 12.9% (66/513). The preoperative characteristics are presented in Table 1. There was no significant difference in sex, body mass index, alcohol consumption and smoking history between the two groups. However, compared with the survivor group, the average age of patients in the non-survivor group was older (57.3 ± 13.6 vs. 53.6 ± 13.5, *p* = 0.036). In addition, more patients in the non-survivor group had preoperative shock (19.7% *vs.* 9.8%; *p* = 0.017) and organ malperfusion (34.8% *vs.* 18.3%; *p* = 0.002). The average fibrinogen level was decreased and the levels of white blood cells, c-reactive protein, D-dimer, troponin T, and brain natriuretic peptide were increased in the non-survivor group compared to the survivor group.

**TABLE 1:** Comparison of preoperative variables.

Variables	Total (*n* = 513)	Non-survivor (*n* = 66)	Survivor (*n* = 447)	*p* Value
Demographic data				
Age (year)	54.1 ± 13.5	57.3 ± 13.6	53.6 ± 13.5	0.036
Male (%)	379 (73.9)	50 (75.8)	329 (73.6)	0.710
BMI (kg/m^2^)	25.8 ± 4.0	26.4 ± 5.0	25.7 ± 3.9	0.337
Smoking (%)	171 (33.3)	25 (37.9)	146 (32.7)	0.401
Drinking (%)	117 (22.8)	15 (22.7)	102 (22.8)	0.987
Time from onset to surgery (h)	14.0 (6.0, 25.5)	13.0 (5.0, 20.3)	15.5 (8.0, 22.0)	0.100
Medical history				
Hypertension (%)	448 (87.5)	60 (90.9)	388 (87.0)	0.370
Diabetes mellitus (%)	19 (3.7)	1 (1.5)	18 (4.0)	0.491
Previous cardiovascular disease (%)	16 (3.1)	3 (4.5)	13 (2.9)	0.707
Cerebrovascular disease (%)	21 (4.1)	3 (4.5)	18 (4.0)	1.000
Marfan syndrome (%)	9 (1.8)	1 (1.5)	8 (1.8)	1.000
COPD (%)	5 (1.0)	1 (1.5)	4 (0.9)	1.000
Atrial fibrillation (%)	9 (1.8)	3 (4.5)	6 (1.3)	0.097
Dialysis (%)	7 (1.4)	0 (0)	7 (1.6)	0.603
Previous cardiac surgery (%)	3 (0.6)	0 (0)	3 (0.7)	0.996
Any malperfusion (%)	105 (20.5)	23 (34.8)	82 (18.3)	0.002
Shock (%)	57 (11.1)	13 (19.7)	44 (9.8)	0.017
Pericardial effusion (%)	416 (82.2)	56 (86.2)	360 (81.6)	0.374
DeBakey type I aortic dissection (%)	412 (80.3)	53 (80.3)	359 (80.3)	0.998
Laboratory tests				
WBC (10^9^/L)	11.2 ± 3.6	12.6 ± 4.1	11.0 ± 3.5	0.001
PLT (10^9^/L)	144.8 ± 71.6	129.3 ± 46.1	147.0 ± 74.3	0.065
CRP (mg/dl)	74.6 ± 63.2	94.2 ± 72.2	71.9 ± 61.5	0.010
D-dimer (ng/ml)	6.2 (3.1, 11.9)	8.9 (4.7, 19.5)	5.9 (3.0, 11.3)	0.008
Albumin (g/L)	36.2 ± 6.8	35.3 ± 7.5	36.3 ± 6.7	0.260
TnT (ng/ml)	0.02 (0.01, 0.08)	0.06 (0.01, 0.14)	0.02 (0.01, 0.08)	0.004
ALT (U/L)	19.0 (14.0, 44.0)	31.0 (15.0, 51.3)	19.0 (14.0, 41.4)	0.570
sCr (μmol/L)	79.0 (61.0, 113.3)	104.0 (71.0. 143.0)	77.0 (60.0, 105.0)	< 0.001
BNP (pg/ml)	83.9 (14.0, 44.0)	110.0 (35.7, 334.5)	80.1 (31.1, 159.0)	0.091
Fibrinogen (g/L)	2.1 ± 1.4	1.7 ± 0.9	2.2 ± 1.5	0.002

Values are mean ± standard deviation, median (interquartile range) or number (percentage).

BMI, body mass index; COPD, chronic obstructive pulmonary disease; WBC, white blood cell; PLT, platelet; CRP, c-reactive protein; TnT, troponin T; ALT, alanine aminotransferase; sCr, serum creatinine; BNP, brain natriuretic peptide.

As shown in Table 2, patients in the non-survivor group had a longer duration of CPB (222.7 ± 147.3 *vs*. 163.4 ± 58.9 min, *p* = 0.002), aortic cross-clamp time (151.9 ± 53.5 *vs*. 137.4 ± 35.2 min, *p* = 0.035), and operation time (8.3 ± 2.8 *vs.* 6.8 ± 1.6 h, *p* < 0.001) compared to patients in the survivor group. In addition, a significantly increased number of patients required concomitant coronary artery bypass grafting after aortic central repair in the non-survivor group compared to the survivor group (12.1% *vs.* 2.5%; *p* = 0.001). Furthermore, an increased amount of red blood cell units was transfused perioperatively to patients in the non-survivor group compared to the survivor group (12.6 ± 4.2 vs. 10.7 ± 4.0, *p* < 0.001).

**TABLE 2:** Comparison of operative variables.

Variables	Total (*n* = 513)	Non-survivor (*n* = 66)	Survivor (*n* = 447)	*p* Value
Cannulation site				
Femoral (%)	192 (37.4)	31 (47.0)	161 (36.0)	0.086
Axillary (%)	69 (13.5)	4 (6.1)	65 (14.5)	0.059
Axillary + femoral (%)	246 (48.0)	31 (47.0)	215 (48.1)	0.864
Concomitant CABG (%)	19 (3.7)	8 (12.1)	11 (2.5)	0.001
Concomitant MVP (%)	5 (1.0)	2 (3.0)	3 (0.7)	0.127
Concomitant TVP (%)	7 (1.4)	1 (1.5)	6 (1.3)	1.000
CPB time (min)	171.0 ± 78.7	222.7 ± 147.3	163.4 ± 58.9	0.002
Aortic cross-clamp time (min)	139.2 ± 38.3	151.9 ± 53.5	137.4 ± 35.2	0.035
DHCA time (min)	26.6 ± 14.2	27.6 ± 15.4	26.5 ± 14.0	0.581
TAR + elephant trunk (%)	155 (30.2)	18 (27.3)	137 (30.6)	0.577
Fenestrated stent (%)	139 (27.1)	21 (31.8)	118 (26.4)	0.355
Distal vessel repair (%)	10 (1.9)	2 (3.0)	8 (1.8)	0.625
Operation time (h)	7.0 ± 1.8	8.3 ± 2.8	6.8 ± 1.6	< 0.001
RBC transfusion (unit)	10.9 ± 4.1	12.6 ± 4.2	10.7 ± 4.0	< 0.001

Values are mean ± standard deviation, median (interquartile range) or number (percentage).

CABG, coronary artery bypass graft; MVP, mitral valvuloplasty; TVP, tricuspid valvuloplasty; CPB, cardiopulmonary bypass; DHCA, deep hypothermic circulatory arrest; TAR, total arch replacement; RBC, red blood cell.

### Complications

Table 3 shows that more complications occurred in the non-survivor group compared to the survivor group including prolonged mechanical ventilation (70.0% *vs.* 24.0%; *p* < 0.001), reintubation (21.9% *vs.* 4.7%; *p* < 0.001), tracheotomy (18.8% *vs.* 2.9%; *p* < 0.001), acute kidney injury (80.3% *vs.* 51.1%; *p* < 0.001), new onset dialysis (62.1% *vs.* 13.2%; *p* < 0.001), cerebral hemorrhage (4.7% *vs.* 0.2%; *p* = 0.007), cerebral infarction (31.3% *vs.* 6.3%; *p* < 0.001), new onset limb ischemia (15.6% *vs.* 1.1%; *p* < 0.001), and lung infection (67.8% *vs.* 37.5%; *p* < 0.001).

**TABLE 3:** Comparison of postoperative variables.

Variables	Total (n = 513)	Non-survivor (n = 66)	Survivor (n = 447)	*p* Value
PMV (%)	149 (29.4)	42 (70.0)	107 (24.0)	< 0.001
AKI (%)	281 (54.9)	53 (80.3)	228 (51.1)	< 0.001
New onset dialysis (%)	100 (19.5)	41 (62.1)	59 (13.2)	< 0.001
Reintubation (%)	35 (6.8)	14 (21.9)	21 (4.7)	< 0.001
Re-exploration for bleeding (%)	20 (3.9)	3 (4.5)	17 (3.8)	1.000
Tracheotomy (%)	25 (4.9)	12 (18.8)	13 (2.9)	< 0.001
Cerebral infarction (%)	48 (9.4)	20 (31.3)	28 (6.3)	< 0.001
Cerebral hemorrhage (%)	4 (0.8)	3 (4.7)	1 (0.2)	0.007
New onset limb ischemia (%)	15 (2.9)	10 (15.6)	5 (1.1)	< 0.001
Paraplegia (%)	22 (4.3)	6 (9.5)	16 (3.6)	0.043
Gastrointestinal bleeding (%)	8 (1.6)	3 (4.7)	5 (1.1)	0.183
Lung infection (%)	207 (41.1)	40 (67.8)	167 (37.5)	< 0.001
SWI (%)	8 (1.6)	0 (0)	8 (1.8)	0.400

Values are mean ± standard deviation, median (interquartile range) or number (percentage).

PMV, prolonged mechanical ventilation; AKI, acute kidney injury; SWI, surgical site infection.

### Blood lactate

**FIGURE 2:**
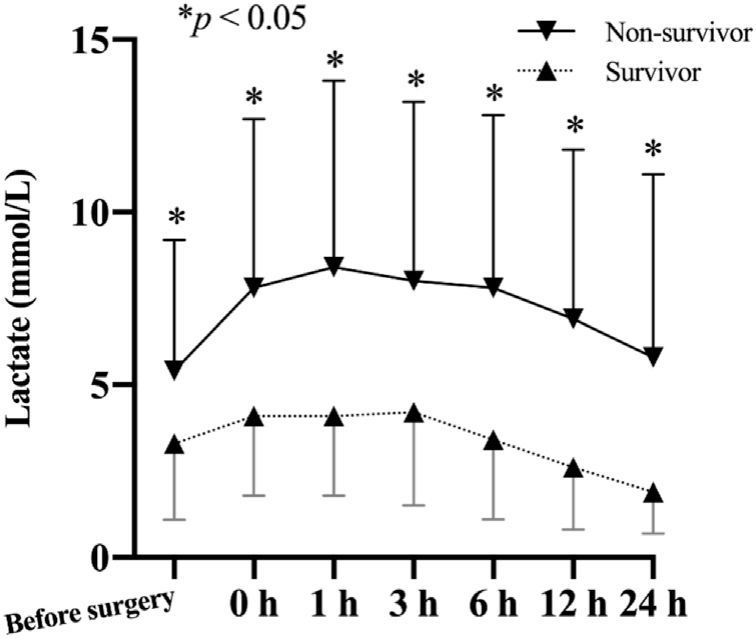
Comparison of lactate levels (mean and standard deviation) of patients from the non-survivor group and the survivor group based on their 30-day mortality.

Figure 2 depicts the change of lactate levels between the two groups. Preoperative lactate levels were significantly higher in the non-survivor group compared to the survivor group (5.4 ± 3.8 mmol/L vs. 3.3 ± 2.2 mmol/L *vs*; *p* = 0.007). After surgery, the difference between two groups persisted and peaked at 1 h after ICU admission.

### Risk factors for 30-day mortality

**FIGURE 3:**
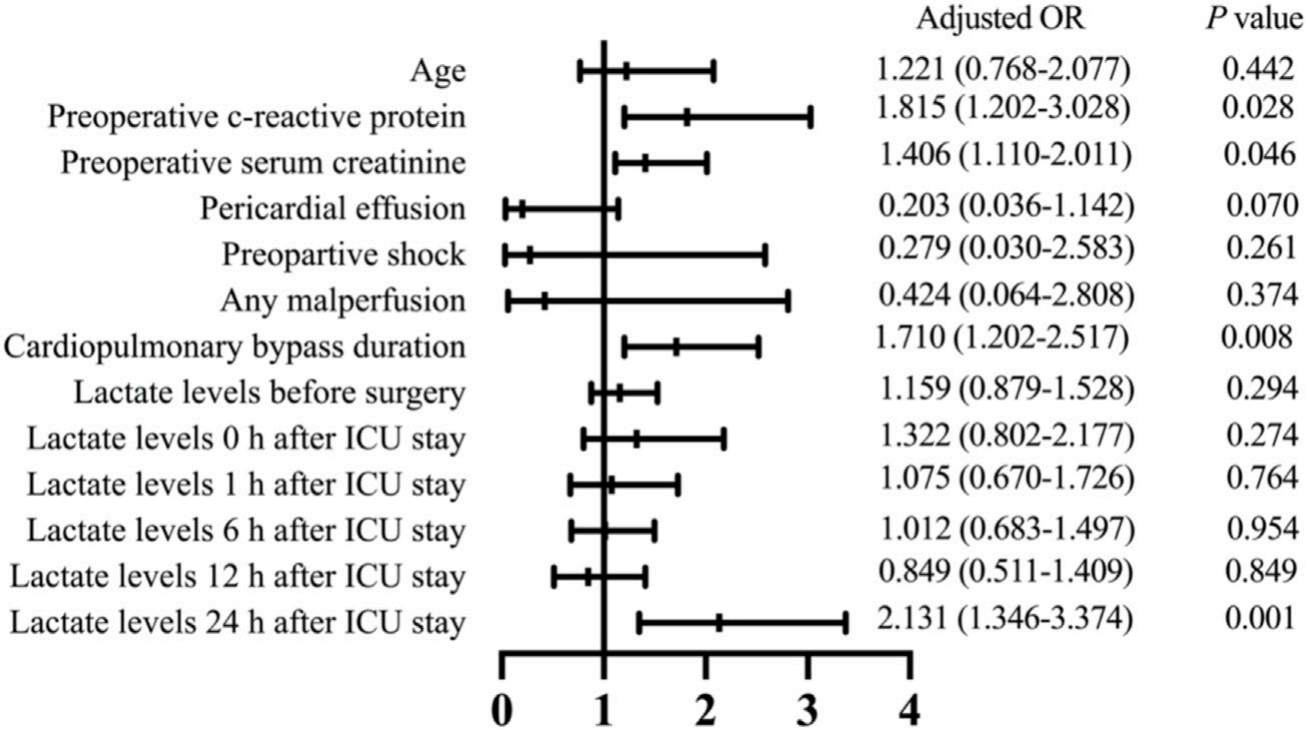
Multivariate analysis results to identify risk factors associated with 30-day mortality in patients who received ATAAD repair surgery. OR, odds ratio; ICU, intensive care unit.

Multivariable logistic risk analysis revealed that preoperative c-reactive protein (odds ratio [OR], 1.815; 95% confidence interval [CI], 1.202–3.028; *p* = 0.028), preoperative serum creatinine (OR, 1.406; 95% CI, 1.110–2.011; *p* = 0.046), CPB time (OR, 1.710; 95% CI, 1.202–2.517; *p* = 0.008), and lactate levels at 24 h after ICU admission (OR, 2.131; 95% CI, 1.346–3.374; *p* =0.001) were independent risk factors for 30-day mortality (Figure 3).

### Receiver operating characteristic analysis

**FIGURE 4:**
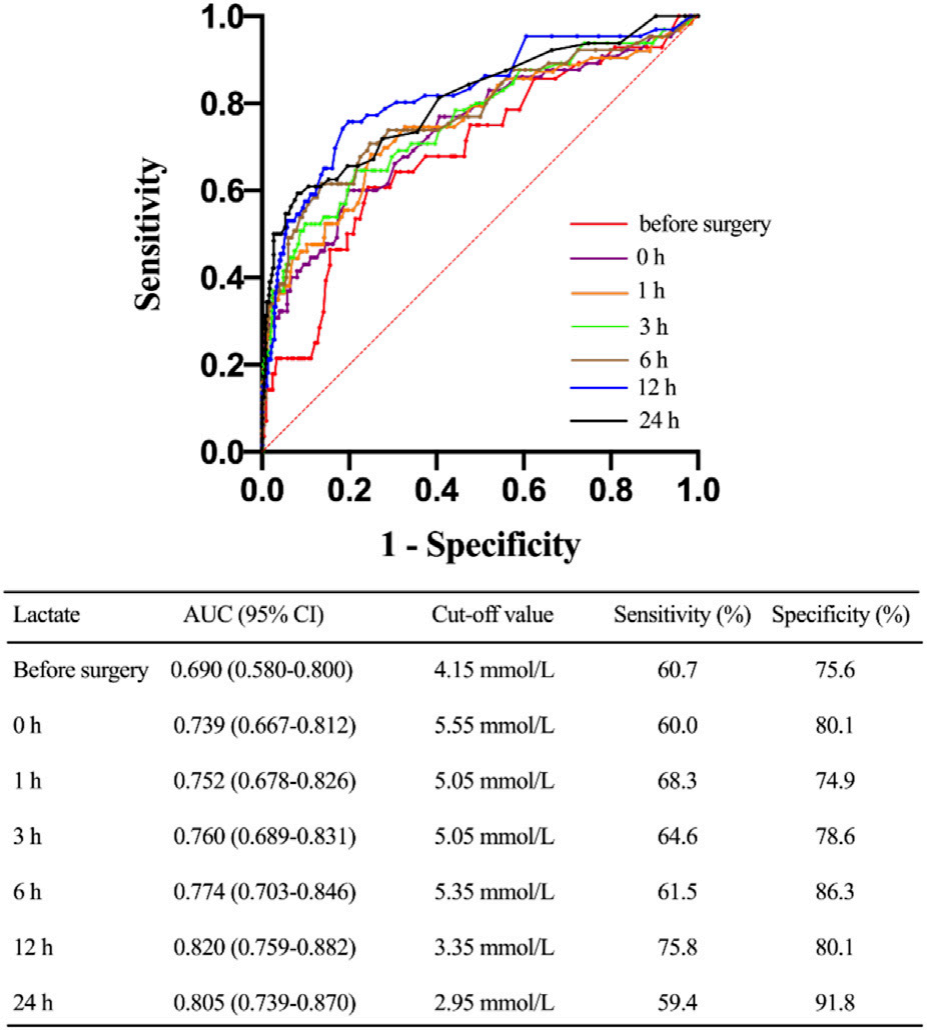
Characteristic of receiving operating curves evaluating the predictive power of different timepoints of lactate levels for 30-day mortality. CI, confidence interval.

**FIGURE 5:**
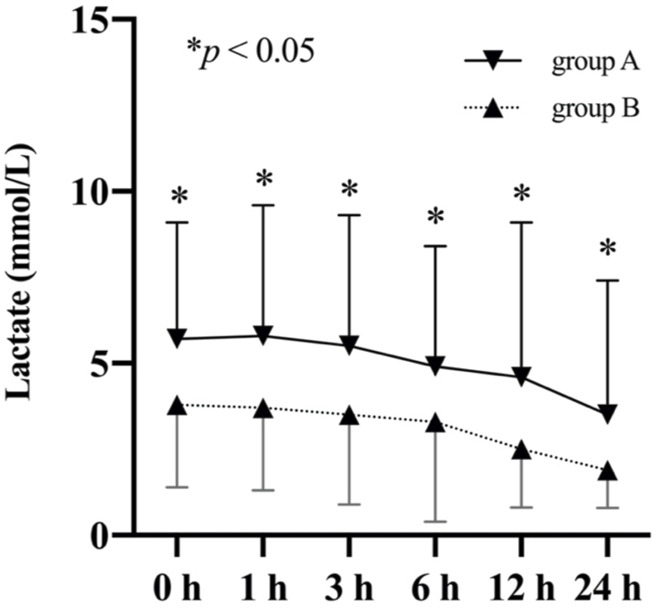
Comparison of lactate levels (mean and standard deviation) of patients stratified based on their preoperative lactate levels.

ROC curves were plotted to identify the proper cut-off value for applying serum lactate level to predict 30-day mortality (Figure 4). The area under the curve (AUC) in relation to lactate level at 12 h after ICU stay was 0.820. A Youden’s index value of 3.35 mmol/L was derived from this curve, giving a sensitivity of 75.8% and a specificity of 80.1%. For lactic acid levels measured at 24 h after ICU stay, the AUC for 30-day mortality was 0.805 with corresponding Youden’s index value of 2.95 mmol/L and a sensitivity of 59.4% while a specificity of 91.8%. For lactate levels before surgery, the AUC for 30-day mortality was 0.690, and the Youden’s index value was 4.15 mmol/L. To test the preoperative lactate value as a categorical dichotomic variable against mortality, we divided the patients into two groups: group A (lactate level >4.15 mmol/L) and group B (lactate level ≤4.15 mmol/L). Data showed that the lactate levels at all postoperative timepoints were significantly higher in group A compared to group B (Figure 5).

## Discussion

Our study suggests that the increasing and dynamic change of lactate levels can be used to predict poor survival with high sensitivity and specificity in patients undergoing ATAAD surgery. The data demonstrated that lactate levels higher than 3.35 mmol/L at 12 h after ICU admission or 2.95 mmol/L at 24 h were associated with an AUC of 0.820 and 0.805, respectively. In addition, an elevated lactate level at 24 h after ICU admission was independently associated with a 2.131-fold increased risk of 30-day mortality in these patients. The results of our study not only proved this association but also sheds light on new perspectives for patient management after ATAAD surgery.

Many risk factors such as hypotension, ischemic events and neurologic abnormalities were identified to be associated with in-hospital mortality in patients who underwent ATAAD surgery (Spirito et al., 2001; Mehta et al., 2002; Czerny et al., 2003; Bekkers et al., 2013). Based on the Penn classification criteria, the risk of having lethal outcomes in patients with ATAAD can be evaluated based on patients’ ischemic events (Olsson et al., 2011; Kimura et al., 2014; Tien et al., 2020). However, many of these risk factors are subjective and require imaging scans that might not be applicable in emergent situations.

It has been shown that serum lactate level is a promising surrogate to evaluate perfusion and has already been used to identify patients with increased risk after undergoing ATAAD surgery. However, conflicting results have been reported regarding its association with disease prognosis. Bennett et al. (Bennett et al., 2017) showed that a cut-off preoperative lactate of 6 mmol/L could predict in-hospital death with an AUC of 0.88. However, this observation was challenged by another study conducted by Zindovic et al. (Zindovic et al., 2018) which demonstrated that preoperative hyperlactemia could not be used to predict disease prognosis. In this study, they found that the AUC for in-hospital mortality in relation to preoperative lactate levels was 0.684, which was similar to our results. However, for lactate levels measured upon ICU admission, Zindovic and others showed the AUC for their association with in-hospital death was only 0.582, which was lower than the value reported in our study. We found that preoperative hyperlactemia was transient in a large portion of their patients, as the levels rapidly declined upon ICU admission. On the other hand, our study showed that the average serum lactate level continued to increase and peaked after ICU admission, which was similar to the dynamic changes reported in a recent study conducted by Gemelli et al. (2022). The discrepancy of dynamic patterns of lactate levels between the two studies might explain these conflicting conclusions. Additional studies are needed to determine how sustained hyperlactatemia after surgery can affect clinical outcomes.

Serum lactate was a rapid and cost-effective alternative clinical marker for aortic dissection severity or malperfusion/ischemia, regardless of timing of presentation. Although the origin of the hyperlactatemia in ATAAD is numerous and diverse, lactate reducing therapy raise the possibility that targeting therapy to reduce or prevent the initial increase in this variable may prevent complications and improve postoperative outcomes. Firstly, when considering hyperlactemia was induced by organ malperfusion, particularly if mesenteric, coronary ischemia or cerebral is present, adjunctive techniques including catheter-based endovascular and even open surgical revascularization may be appropriate (Stewart and Chikwe, 2015; Li et al., 2016). Secondly, the inflammatory response also played a vital role in the postoperative hyperlactemia development. Mini-CPB could lead to fewer alterations of microperfusion and improve outcomes in patients with longer procedures (Donndorf et al., 2012). In addition, reducing the use of catecholamines during cardiac surgery could lead to decreased lactate levels owing to their action on oxidative glucose metabolism (Bellomo, 2002).

Using a single lactate level measured at a single time point to evaluate a patient’s condition could be misleading, as mild or moderate elevations are often difficult to interpret. Alternatively, serial measurements during the first 24 h after ATAAD surgery may be a more reliable predictor of 30-day mortality as seen in our study. It is important to note that only lactate levels measured at 24 h after ICU admission, but not other earlier time points, were identified as a risk factor for mortality. There may be several explanations for this observation. One hypothesis is that the elevated lactate levels at earlier time points might be due to inflammatory responses rather than reflecting systemic perfusion. Furthermore, many other conditions can contribute to early elevation of lactate levels after surgery including hyperglycemia, prolonged CPB, inotropic and vasopressor effects of epinephrine (which is often required in the post-surgery period), and elevated secretion of endogenous catecholamines (O'Connor and Fraser, 2012).

Our study showed that increased CPB time was another independent risk factor for short-term mortality. Recent studies indicated that increased CPB time could induce postoperative hyperlactemia in patients undergoing ATAAD surgery (Wang et al., 2021). It has been well known that microcirculation can affect the oxygen supply to cells. Greenwood et al. (2021) demonstrated that microcirculation was often impaired in cardiac surgery with CPB and was associated with postoperative hyperlactemia. In addition, liver clearance of lactate can be reduced secondary to the use of anesthetics and lower temperatures during CPB which might reduce the rate of cellular metabolism (Takala et al., 1996; Raper et al., 1997).

Malperfusion syndromes identified upon hospital admission was identified in a previous study to be associated with poor outcomes after surgery for ATAAD (Czerny et al., 2015). Our data showed the organ malperfusion rate and the preoperative lactate levels were higher in the non-survivor group. However, the association between preoperative malperfusion syndromes and poor outcomes was not observed in our logistic model. This discrepancy could be explained by the fact that prompt surgery was often performed for these patients in our center. Under such a policy, the prognosis of these patients following emergent surgery can be similar to those who did not present with malperfusion syndromes upon admission (Xue et al., 2021). With recent advances of surgical techniques and equipment, the overall perioperative mortality and long‐term survival rate of ATAAD patients with organ malperfusion has been greatly improved.

## Limitations

Our study has several limitations. First, all patients enrolled in this study were retrospectively collected from a single center, and therefore might not be representative of all AATD patients undergoing emergent surgery. Second, the causes of elevated lactate levels were not further stratified due to limitations of our database. Furthermore, the mortality rate reported in our study was relatively low compared to previous studies which might underestimate the statistical power of other potential risk factors.

## Conclusion

In conclusion, our data indicates that dynamic lactate levels after ATAAD surgery provides important prognostic data and is associated with increased postoperative mortality.

## Data Availability

The raw data supporting the conclusions of this article will be made available by the authors, without undue reservation.

## Abbreviations

ATAAD, acute type A aortic dissection; ICU, intensive care unit; AUC, the area under the curve; ROC, receiver operating characteristic; CPB, cardiopulmonary bypass; OR, odds ratio; CI, confidence interval.
